# 2772. Isolation and Characterization of Lytic Bacteriophages Targeting Clinical Isolates of genus *Enterobacter*

**DOI:** 10.1093/ofid/ofad500.2383

**Published:** 2023-11-27

**Authors:** Yanling He, Zhiyong Zong

**Affiliations:** West China hospital, Chengdu, Sichuan, China; West China Hospital, Chengdu, Sichuan, China

## Abstract

**Background:**

*Enterobacter* strains are part of the human intestinal commensal microflora and important pathogens of hospital-acquired infections,such as bacteremia, lower respiratory tract infections, and urinary tract infections.

In an era of increasing antibiotic resistance, bacteriophage therapy is one way to combat bacterial pathogens considering its unique merits,such as self-replicating, self-limiting, safe, and highly specific.

**Methods:**

Bacteriophages were isolated from water samples collected from Jinjiang River in Chengdu, PR China and sewage water from a hospital of the same city.Twenty carbapenem-resistant clinical isolates represent common species of *Enterobacter* were used to screen phages. Biological characteristics of all isolated phages were analyzed including host range determination with a panel of 50 *Enterobacter* strains and planktonic bacterial lysis assay of the host cells.All phages were subjected to genome sequencing.

**Results:**

Twenty-three lytic bacteriophages were isolated and most of which belong to three families,*Straboviridae*(n=17). Two phages are respectively members of *Autographiviridae*(n=1) and *Casjensviridae*(n=1) as novel genera.Four phages could not be matched into any known classification.Most phages could lyse multiple isolates of genus *Enterobacter* as well as one isolate of genus *Huaxiibacter*.

The determination of lytic activity in vitro revealed that most phages are able to Inhibit bacterial growth for 4-24 hours.

taxonomic information of bacteriophages against Enterobacter

**Figure:**
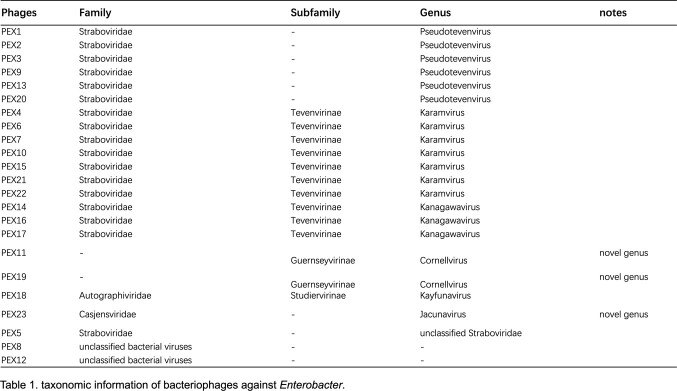

Partial lytic profiles of bacteriophages against Enterobacter

**Figure:**
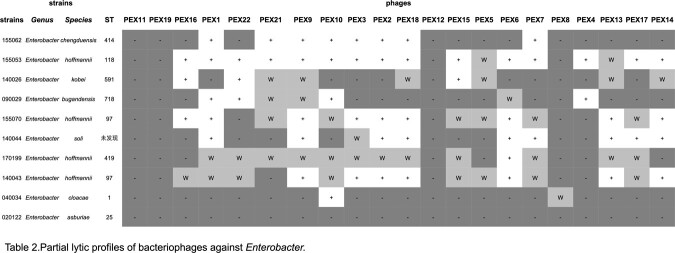

Planktonic cell lysis curve

**Figure:**
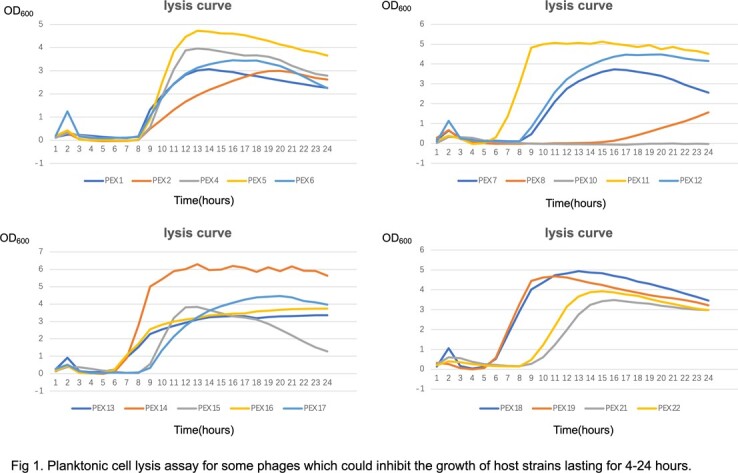

**Conclusion:**

*Enterobacter*-targeting phages has a wide lysis spectrum and some phages were significantly inhibited for an extended period of more than 24 hours.These results indicate the potential value of bacteriophages against *Enterobacter* infection in clinical treatment.

**Disclosures:**

**All Authors**: No reported disclosures

